# Effects of Laser Acupuncture on Longitudinal Bone Growth in Adolescent Rats

**DOI:** 10.1155/2013/424587

**Published:** 2013-08-06

**Authors:** Mijung Yeom, Sung-Hun Kim, Bina Lee, Xiuyu Zhang, Hyangsook Lee, Dae-Hyun Hahm, Youngjoo Sohn, Hyejung Lee

**Affiliations:** ^1^Acupuncture and Meridian Science Research Center, College of Korean Medicine, Kyung Hee University, Seoul 130-701, Republic of Korea; ^2^Department of Science in Korean Medicine, College of Korean Medicine, Kyung Hee University, Seoul 130-701, Republic of Korea

## Abstract

Longitudinal bone growth is the results of chondrocyte proliferation and hypertrophy and subsequent endochondral ossification in the growth plate. Recently, laser acupuncture (LA), an intervention to stimulate acupoint with low-level laser irradiation, has been suggested as an intervention to improve the longitudinal bone growth. This study investigated the effects of laser acupuncture on growth, particularly longitudinal bone growth in adolescent male rats. Laser acupuncture was performed once every other day for a total of 9 treatments over 18 days to adolescent male rats. Morphometry of the growth plate, longitudinal bone growth rate, and the protein expression of BMP-2 and IGF-1 in growth plate were observed. The bone growth rate and the heights of growth plates were significantly increased by laser acupuncture. BMP-2 but not IGF-1 immunostaining in growth plate was increased as well. In conclusion, LA promotes longitudinal bone growth in adolescent rats, suggesting that laser acupuncture may be a promising intervention for improving the growth potential for children and adolescents.

## 1. Introduction

Short stature in childhood and adulthood is one important concern. Most of people desire to get taller due to the thought that long height may help to improve the physical appearance and attractive personality or that to be unusually short may have social disadvantages. In fact, short stature (height) is one of major causes of concern and anxiety to children, adolescents, and parents [1]. For this reason, interest in the growth promotion in childhood and adolescent periods is increasing.

An improvement in the height is significantly associated with the bone growth in length. Bone growth in the length occurs at the growth plate by endochondral ossification, a two-step process in which cartilage is first formed and then remodeled into hard calcified bone [2, 3]. The growth plates are composed of three functionally and structurally distinct layers: the resting, the proliferative, and the hypertrophic zones. At this site, growth plate chondrocytes proliferate, differentiate into mature hypertrophic cells, synthesize the typical extracellular matrix, and form cartilage. The newly formed cartilage is then calcified and converted into hard bone, resulting in bone elongation [4]. 

Growth hormone (GH) therapy has been often considered as a treatment option to increase height in children. Its effectiveness is, however, still doubtful. Although GH therapy for short children seems effective in increasing growth and final height, individual responses in the therapy are highly variable and the mean final height in treated individuals remains relatively short when compared with normal stature [5, 6]; health-related quality of life in children treated with GH has been the same, or even worse [7]. Although GH treatment in children has been known to be generally safe, its significant side effects such as benign intracranial hypertension, slipped capital femoral epiphysis, scoliosis, features of acromegaly, pancreatitis, and the increase in cancers have been reported [8]. Also, the long-term risks of prolonged GH treatment of children remain still unknown. Hence, there is a strong need for alternative therapies that are safe and effective for stimulating the growth plate and finally increasing height of short children. 

According to traditional beliefs, disease results from stagnation of the flow of the body's vital energy, “Qi”; acupuncture stimulation at acupoint would help restore the flow and then promote the homeostasis of the Qi, reestablishing the body function to normal [9]. Being considered a relatively simple, inexpensive, and safe treatment compared to other conventional interventions, acupuncture is regarded as a nonpharmacological alternative to GH treatment; however, since the most children are afraid of needles, they would be unwilling to undergo acupuncture and the parents may hesitate to expose them to additional pain and stress [10]. For this reason, laser acupuncture is emerging as an alternative to needle acupuncture [11].

Laser acupuncture is one of interesting modalities of low-level laser therapy (LLLT), a noninvasive form of phototherapy, which is defined as the stimulation of traditional acupoints with low-intensity, nonthermal laser irradiation [12, 13]. The noninvasive and painless nature of treatment makes it useful for particularly providing children with holistic healthcare [12]. Although using low-level, nonthermal laser irradiation instead of needles, it shares the same principles of the traditional acupuncture and proves equal biological effect as needle acupuncture [14]. To our knowledge, studies demonstrating the effect of acupuncture including laser acupuncture on longitudinal bone growth in adolescent rats have previously not been reported.

The purpose of the present study was to investigate the effects of laser acupuncture on longitudinal bone growth in adolescent rat.

## 2. Materials and Methods

### 2.1. Animals

Male Sprague–Dawley rats aged 2 weeks were purchased with their mothers from Samtaco Co. (Osan, Korea). Animals were housed in an animal room maintained at 20° ± 2°C with lighting (07:00–19:00). They had free access to water and food. After a 1-week acclimation period, the animals weighing approximately 40 g were used in the experiments. All studies were conducted in accordance with the National Institutes of Health (NIH) guidelines and were approved by the Institutional Animal Care and Use Committee of Kyung Hee University.

### 2.2. Experimental Schedules

Rats were randomly divided into two groups (*n* = 10 for each group): control group (CONT) and laser acupuncture treatment group (LA). The laser acupuncture was applied once every other day between 9:00 and 10:00 am, started on day 0, and continued until day 18. Body weight was recorded just before the acupuncture treatment. Nose-to-tail length was measured on day 18. For determining longitudinal bone growth rate, tetracycline (15 mg/kg body weight) was administrated by intraperitoneal injection on days 13 and 16. On day 19, the rats were anaesthetized with an overdose of pentobarbital (100 mg/kg body weight) and the tibias were removed and fixed in 10% buffered formalin (pH 7).

### 2.3. Laser Acupuncture

Laser acupuncture was performed by laser stimulation using Lapex-2000 (Meridian Medical Inc. Vancouver, Canada), a semiconductor-based low level laser therapy (LLLT) device emitting a cold red laser (635–680 nm/40 mW). Before laser acupuncture was applied, both hind limbs were shaved with an electric clipper, paying attention to not hurt the skin. Acupoints ST36 (Zusanli) and SP6 (Sanyinjiao) commonly used in the treatment of growth stimulation were selected in this study [15–17]. Each acupoint of rats lightly restrained by hands without anesthesia was stimulated, bilaterally, for 30 seconds (energy density of 1.2 W/cm^2^) by holding a laser probe with a spot size of the laser of 3 mm in diameter in contact with, and perpendicular to, the acupoints; each treatment session lasted 120 seconds. Control rats were sham operated with the inactivated probe which was turned off. Acupuncture was performed by one of the authors who has a recognized training in traditional Chinese medicine. 

### 2.4. Detection of Longitudinal Bone Growth Rate

The bones of each rat were labeled by administering tetracycline before rats were sacrificed (see “Section 2.2” in detail). Longitudinal bone growth rate was evaluated in undecalcified bone. The tibias fixed in 10% buffered formalin (pH 7) were cryopreserved in 30% sucrose solution and sectioned longitudinally at a thickness of 40 *μ*m using a microtome (Leica Microsystems, Bensheim, Germany). The sections were mounted on collagen-coated glass slides and observed by a confocal fluorescence microscope (FLUOview FV10i; Olympus, Tokyo, Japan) to measure the gap distance between the fluorescent bands of the metaphysis of the proximal tibia. The distance between the bands of tetracycline-labeled bone was measured from the densest part of each band rather than from the edges, which were irregular and more difficult to define. The measurements were performed using FV10-ASW 2.0 microscopy software (Olympus). The distance between the bands is the amount that the tibia has grown by the activity of the proximal epiphyseal cartilage during the two days between injections. Longitudinal bone growth rate was calculated as the interlabel width divided by the number of days between the tetracycline injections. 

### 2.5. Histomorphometrical Evaluation

The fixed tibia specimens were decalcified in a histologic decalcifying agent (Calci-Clear Rapid; National Diagnostics, Atlanta, USA), dehydrated through a graded ethanol series, cleared in xylene, and processed for embedding in paraffin with routine protocols. Four *μ*m-thick sections were cut using a rotatory microtome (Finesse 325; Thermo Shandon, Cheshire, UK), mounted on collagen-coated glass slides, and subsequently stained with toluidine blue. Slides were viewed at a magnification of 100×, and images of the entire growth plate were taken with BX51 microscope (Olympus). Histomorphometry of growth plate was based on the zone definitions [18]. For morphometry, the heights of the resting, proliferative, and hypertrophic zones in growth plate as well as the length of the growth plate were measured as indicated in Figure 2(a) using DP2-BSW software (Olympus). At least 5 measurements per sample were taken at three different locations by two observers blind to the experimental group. Each zone of the growth plate was also scored on a scale of 0–3 based on the number of chondrocytes within the same defined region: 0 = absent, 1 = moderate, 2 = discreet, and 3 = intense [19].

### 2.6. Immunohistochemistry 

After decalcified and paraffin-embedded, the bones were sectioned at a thickness of 4 *μ*m using a rotatory microtome Finesse 325 (Thermo Shandon). For the detection of bone morphogenetic protein-2 (BMP-2) and insulin-like growth factor-1 (IGF-1) in the growth plate, the deparaffinized sections were immersed in 0.01 M sodium citrate buffer (pH 6.0) for 40 min for antigen retrieval and incubated with rabbit anti-BMP-2 antibody (1 : 200 diluted; Novus Biologicals, Littleton, USA) or mouse anti-IGF-1 antibody (1 : 200 diluted; Novus Biologicals) at 4°C overnight. After washing, sections were processed with the avidin-biotin-peroxidase complex (Vectastain Elite ABC kit; Vector Labs, Burlingame, USA) including the appropriated secondary anti-rabbit or anti-mouse IgG (biotinylated, 1 : 200 diluted; Vector Labs) and developed with the DAB peroxidase substrate kit (Vector Labs). Slides were counterstained with hematoxylin. Staining was completely absent in identical tissue sections in which the primary antibody was omitted (data not shown). Images were taken with BX51 microscope (Olympus).

### 2.7. Statistical Analysis

All data were presented as the means ± standard error of the mean (SEM) for each group. Statistical significance was calculated with Student's *t*-test using Graphpad Prism 5 software package (GraphPad Software, San Diego, USA). Differences were considered significant when *P* < 0.05.

## 3. Results and Discussion

**Figure 1:**
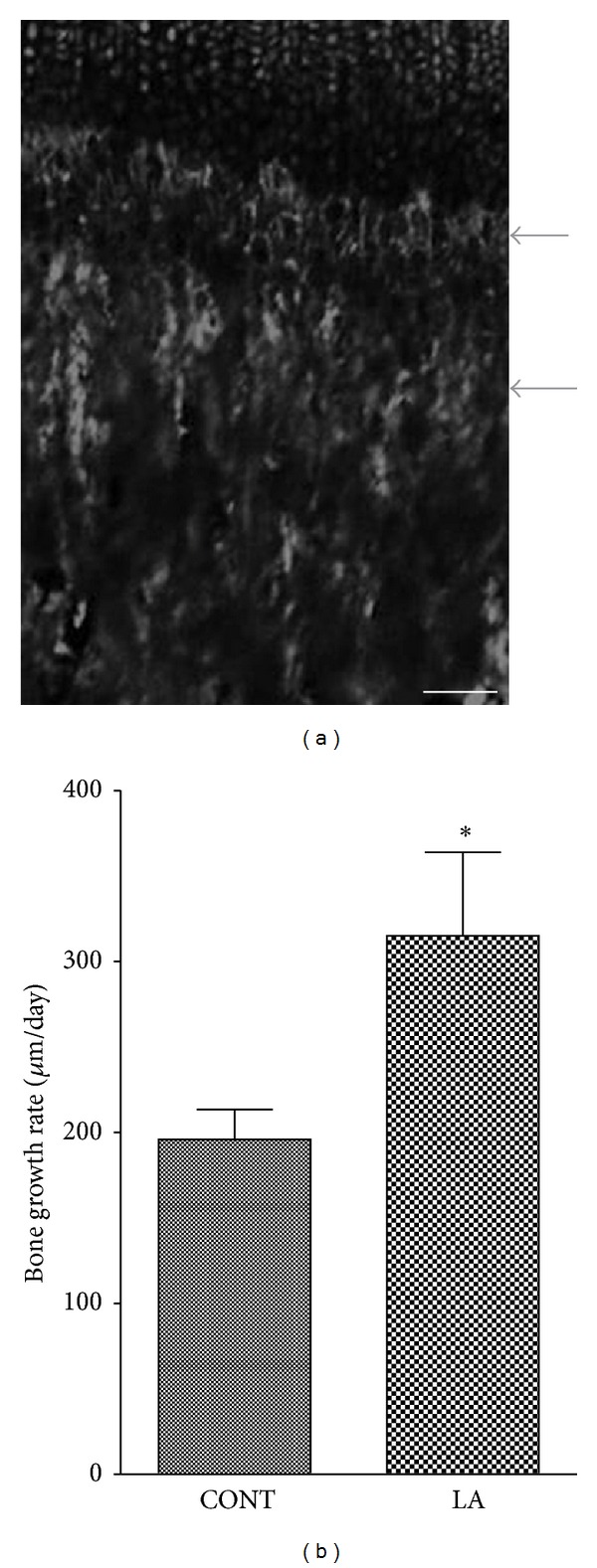
Effects of laser acupuncture on the longitudinal bone growth of the proximal tibia. Longitudinal bone growth rate was determined by tetracycline labeling. Representative fluorescent image of a longitudinal section of the proximal tibia showing tetracycline labeling (a) is shown. Arrows indicate two fluorescent lines representing the two injections of tetracycline. Scale bar = 100 *μ*m. Longitudinal bone growth rate (b) was calculated by means of tetracycline labeling (*μ*m/day). CONT, control group; LA, laser acupuncture group. Each value is the mean ± SEM. *P < 0.05 versus control group.

Neither Body weight nor the nose to tail length was different between control and LA-treated groups (data not shown). To evaluate the longitudinal bone growth rate, tetracycline labeling was used to stain newly formed bone. Tetracycline binds to free calcium and gets deposited in newly deposited bone, causing staining and fluorescence under ultraviolet illumination. The tetracycline administered to rats formed two fluorescent lines corresponding to the two injections (Figure 1(a)). The longitudinal bone growth rate in normal adolescent rats was 195.9 ± 17.5 *μ*m/day and laser acupuncture was shown to promote bone growth, increasing the rate to 315.1 ± 48.8 *μ*m/day (Figure 1(b)). About 200 *μ*m/day of the longitudinal bone growth rate in control group is in accordance with the results reported previously [20].

**Figure 2:**
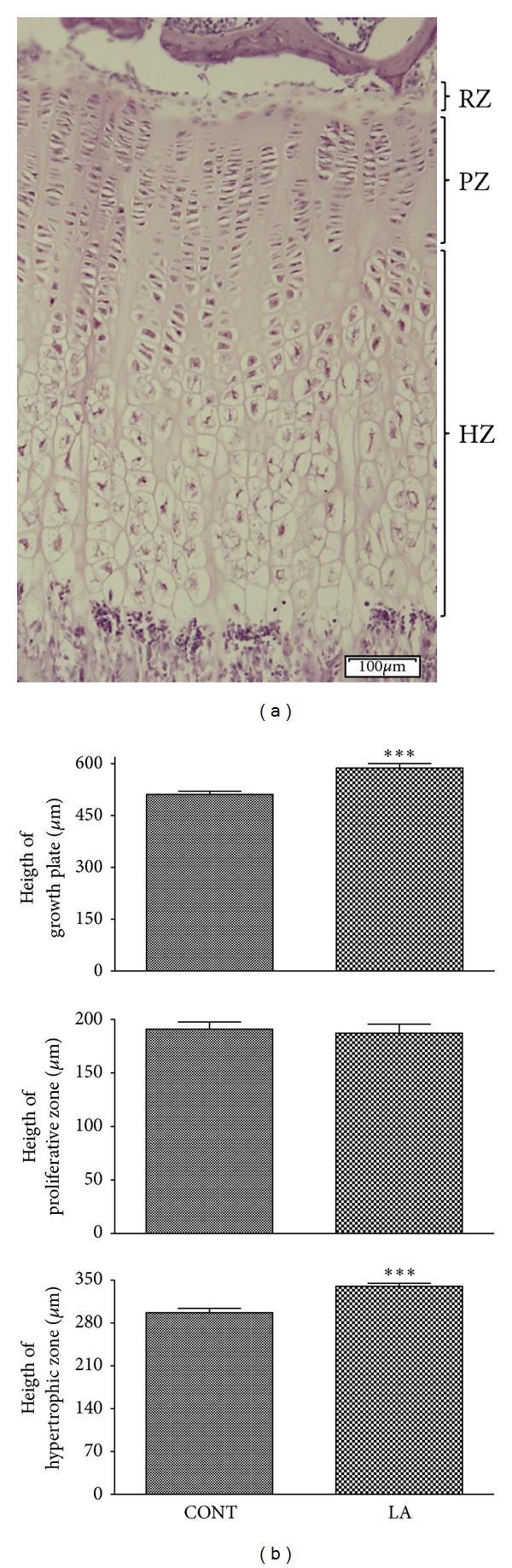
Effects of laser acupuncture on zone-specific growth within the growth plate. Representative proximal tibia section from normal rat (a) is shown. The tissues were stained with toluidine blue in which cartilage is pink. Resting zone (RZ), proliferative zone (PZ), and hypertrophic zone (HZ) are designated. Scale bar = 100 *μ*m. The length of the entire growth plate and the length of the proliferation zone and the hypertrophic zone were measured separately using image processing software (b). CONT, control group; LA, laser acupuncture group. Each value is the mean ± SEM. ***P < 0.005 versus control group.

The increase in body length is mostly due to the longitudinal bone growth, which is a reflection of the synchronized processes of chondrogenesis (the proliferation and differentiation of chondrocytes in the growth plates) and cartilage ossification (calcification at the metaphysis) [21]. Interestingly, laser acupuncture significantly increased longitudinal bone growth. As the height of growth plate was correlated with the body growth rate [22], the heights of the proximal tibia growth plate were measured. The height of proximal tibia growth plate in normal adolescent rats was 511.3 ± 9.2 *μ*m. Following laser acupuncture treatment, growth plate height increased to 587.5 ± 13.0 *μ*m (Figure 2(b) top). The growth plate is composed of three layers: the resting, proliferative, and hypertrophic zones [18].

**Figure 3:**
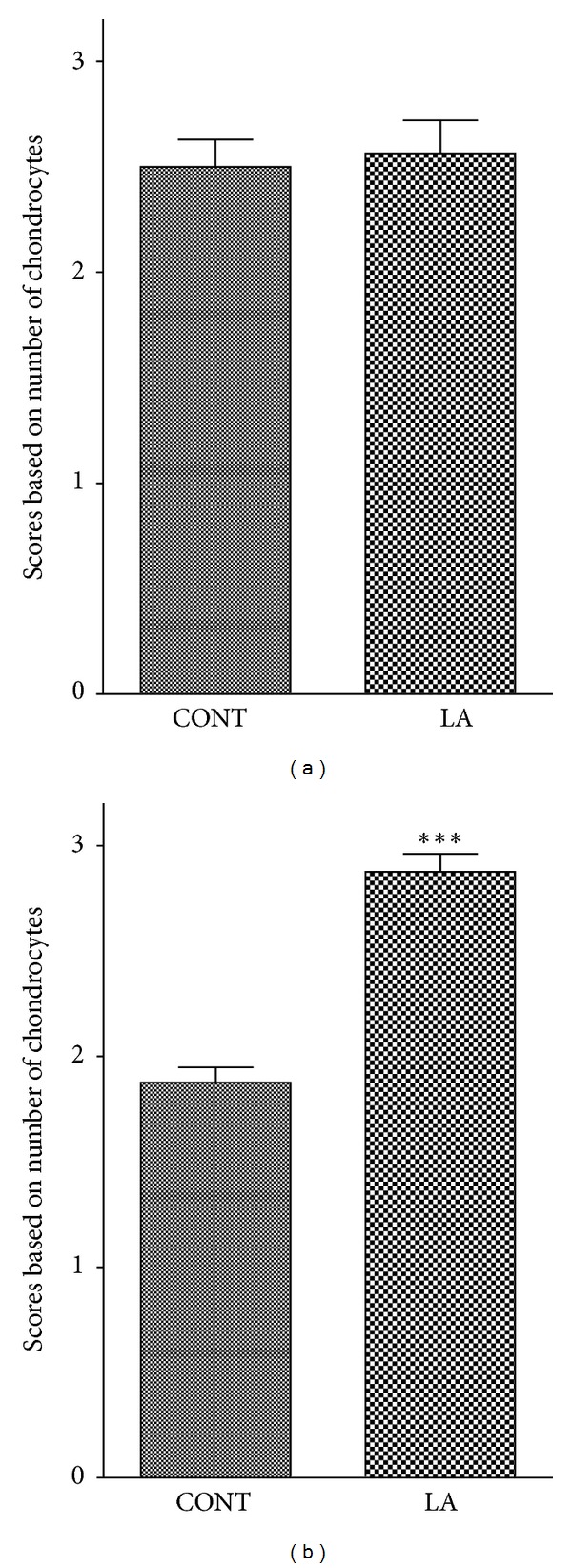
Effects of laser acupuncture on cell density in the proliferative and hypertrophic zones of the growth plate. The proliferative (a) and hypertrophic (b) zones of the growth plate were scored in accordance with the number of chondrocytes within the same defined region. CONT, control group; LA, laser acupuncture group. Each value is the mean ± SEM. ***P < 0.005 versus control group.

The heights of proliferative and hypertrophic zones were also measured since the resting zone was almost absent in the vast majority of animals. Contrary to our expectation that LA may increase the height of both proliferative and hypertrophic zones, it only affected the hypertrophic zone; the height of hypertrophic zone was slightly but significantly higher in LA group as compared to control group (Figure 2(b) bottom), but that of proliferative zone was not different (Figure 2(b) middle). The proportion (%) of each zone in the growth plate was similar in control and LA-treated rats (data not shown). In addition, the scores were determined for the cell density in the proliferative and hypertrophic zones of the growth plate, which was in accordance with the morphometric analysis. The cell density in the hypertrophic zone was significantly higher in laser acupuncture-treated group compared to control group (Figure 3(b)), but not different in the proliferative zone (Figure 3(a)). Although the rate of new cell production and matrix production in the proliferative zone is an important factor in bone formation, the hypertrophic zone plays a key role as well. Hypertrophic chondrocytes generated by terminal differentiation of chondrocytes in the proliferative zone cease dividing and then enlarge, contributing substantially to the growth process such as the initiation of ossification [23]. The amount of enlargement of the hypertrophic cells in the direction of growth together with a change in the height of the hypertrophic zone is important factors in the difference in the growth rate. Because chondrocytes no longer proliferate after early hypertrophy, the number of cell layers in the hypertrophic zone only reflects the time spent by the cells in this region. Therefore, the increase of chondrocyte numbers in the hypertrophic zone might, however, be not sufficient to affect the total length of the body.

**Figure 4:**
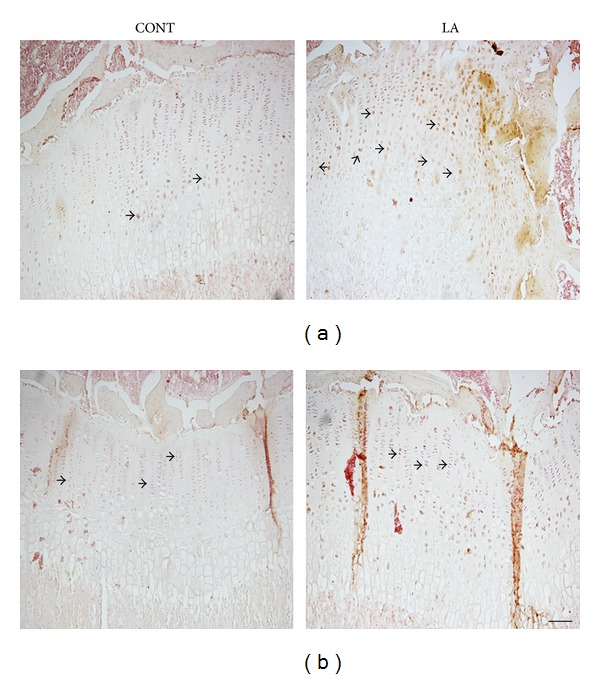
Effects of laser acupuncture on BMP-2 and IGF-1 expression in the growth plate. Representative images of immunostaining for BMP-2 (a) and IGF-1 (b) in proximal tibial growth plates of control and laser acupuncture-treated rats are shown. Arrows indicate brown positive staining with BMP-2 or IGF-1. CONT, control group; LA, laser acupuncture group. Scale bar = 100 *μ*m.

Next, the expression of BMP-2 and IGF-1 was investigated in the growth plate using immunohistochemistry. BMPs play important roles in regulating growth plate chondrogenesis and longitudinal bone growth as well as embryonic skeletal development. BMP-2 stimulates chondrocyte proliferation in the proliferative zone of the growth plate and also causes an increase in chondrocyte hypertrophy [24]. BMP-2 immunostaining was intense in hypertrophic zone but weak in the proliferative zone of the growth plate. As expected, BMP-2 expression particularly in hypertrophic chondrocytes of the growth plate was increased in the laser acupuncture-treated group compared with the control group (Figure 4(a), arrow denotes brown staining indicative of BMP-2 expression). IGF-1 is an important factor to augment longitudinalbone growth by stimulating growth plate chondrocyte proliferation [25]. IGF-1 immunostaining was relatively higher in the proliferative zone than the hypertrophic zone. But, as expected, IGF-1 expression was similar between control and laser acupuncture-treated rats (Figure 4(b), arrow denotes brown staining indicative of IGF-1 expression).

Although the mechanism of laser acupuncture is not completely understood, the following effects of LLLT are known: increases of cell growth, cell regeneration, and cellular activity [26]. This may help to explain the positive mechanism of laser acupuncture on longitudinal bone growth. Our data in this study are actually in accordance with the effects of LLLT on cell activity; the stimulation of acupoint with low-level laser increased the height and the chondrocyte numbers in the hypertrophic zone and thus promoted the longitudinal bone growth rate. Also, the regulation by systemic hormones such as GH and IGF-1 on longitudinal growth and final height is well known [27]. Therefore, another possibility to explain the action mechanism of LA on bone growth is that LA may enhance the bone growth in length through the regulation of the hormone system. Further studies are necessary for better understanding of the effects of LA on bone growth.

A few of studies have demonstrated the effects of LLLT on epiphyseal growth, but results are controversial. Some findings support that LLLT has the effects to induce chondrogenesis in vitro [28] and to improve cartilage structure in vivo [29, 30], while others prove that LLLT has no [31] or even negative [19] effect on bone growth. These differences require further study.

In conclusion, laser acupuncture induces the longitudinal bone growth in adolescent rats through the induction of BMP-2 and IGF-1, suggesting that this treatment may have a clinical potential in promoting longitudinal bone growth in children.
